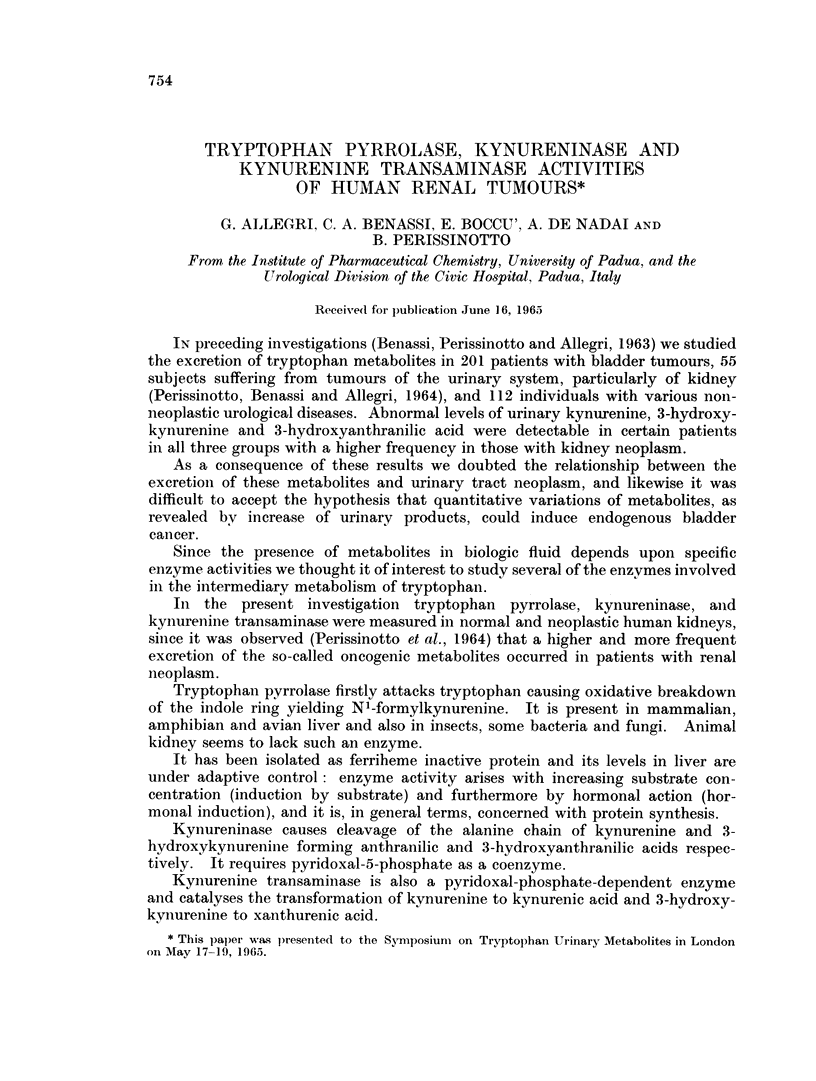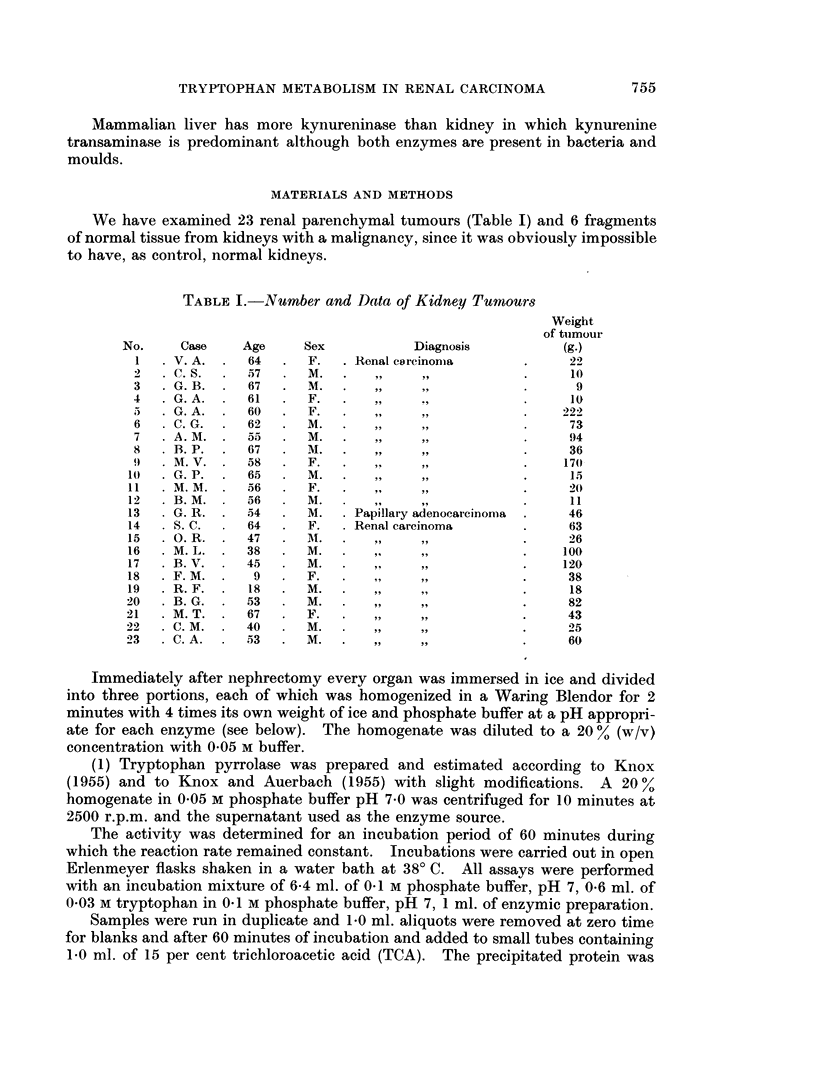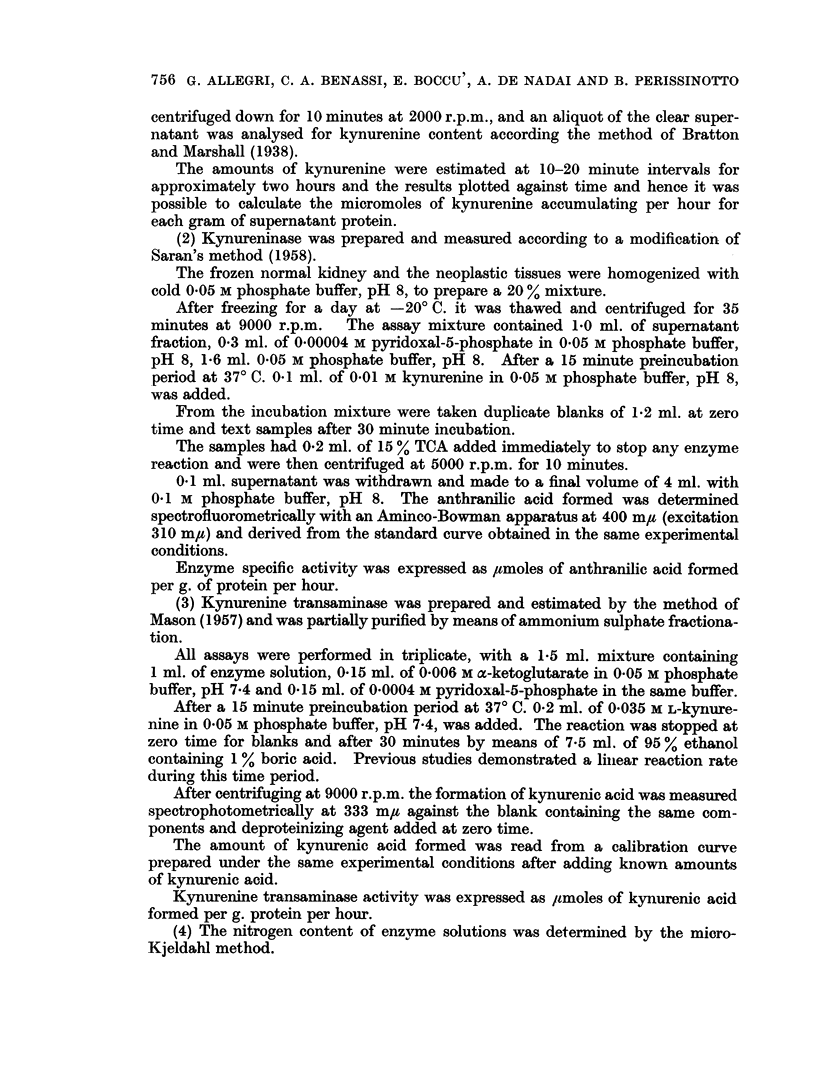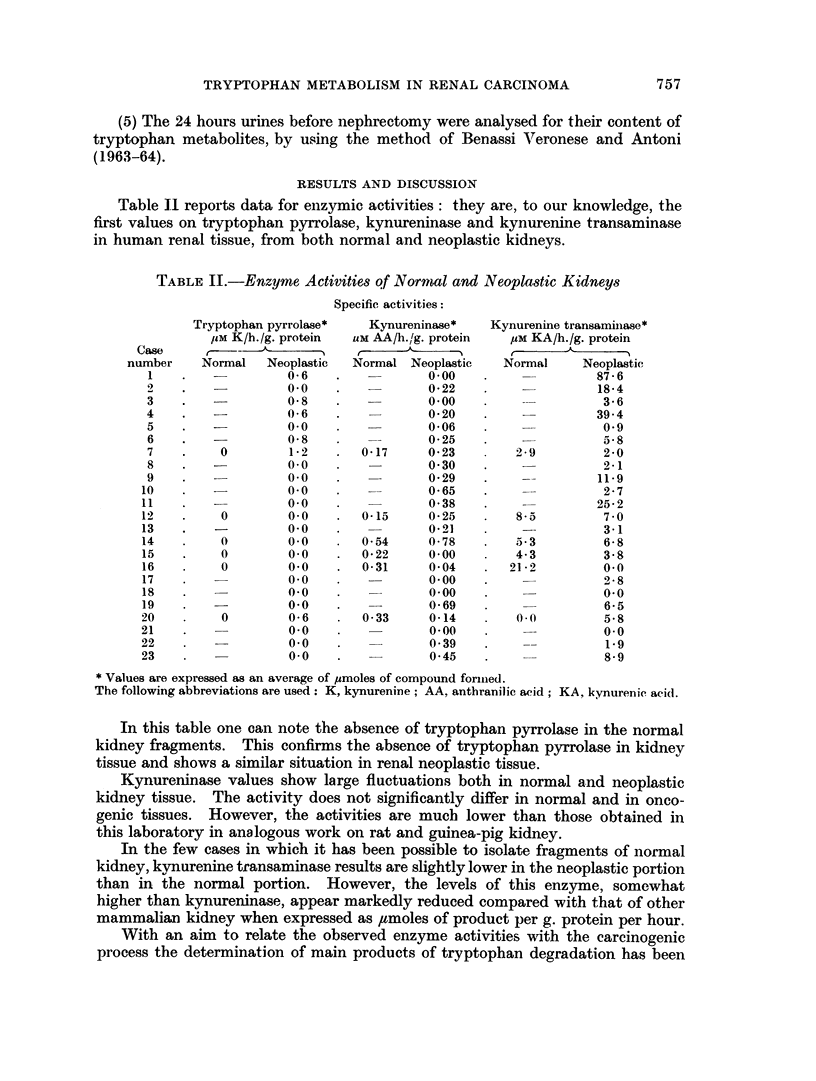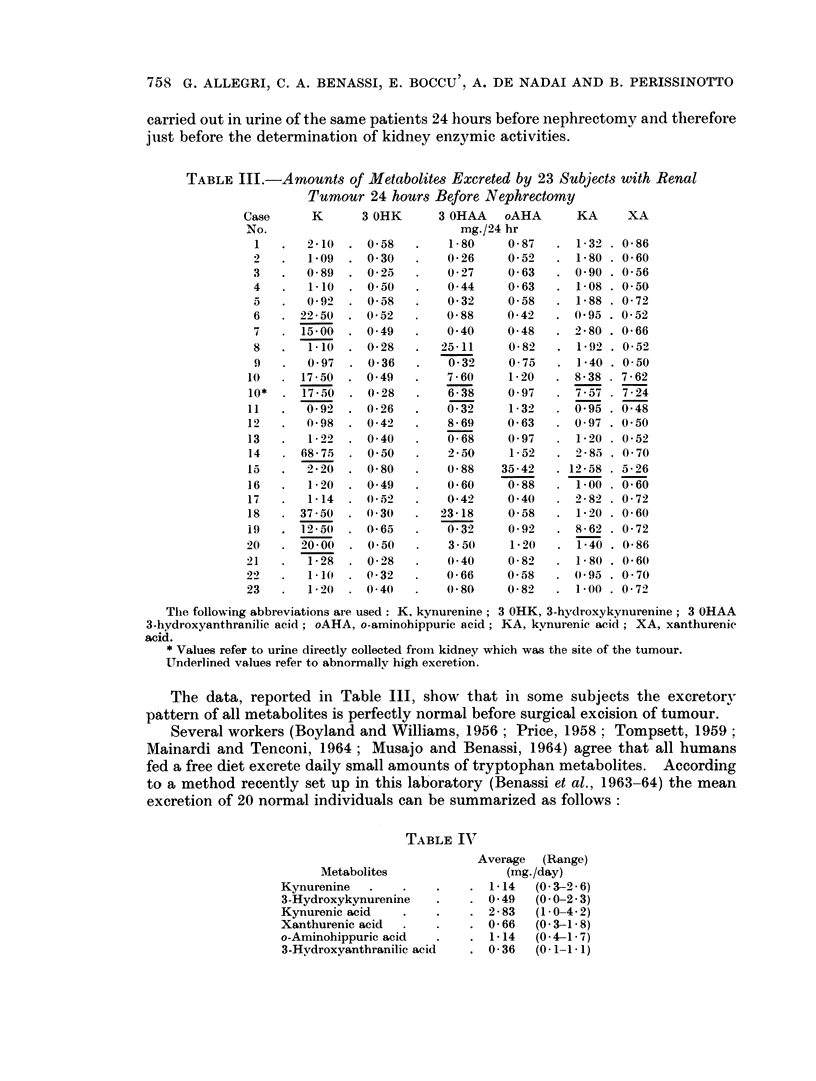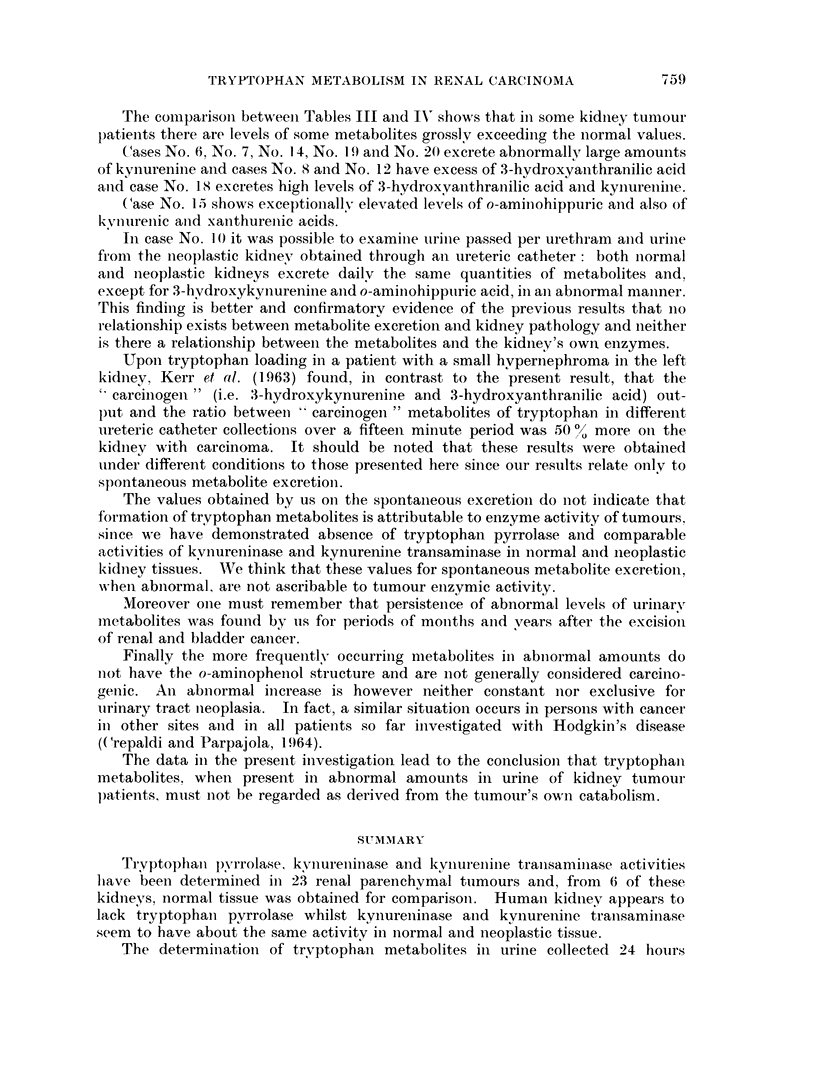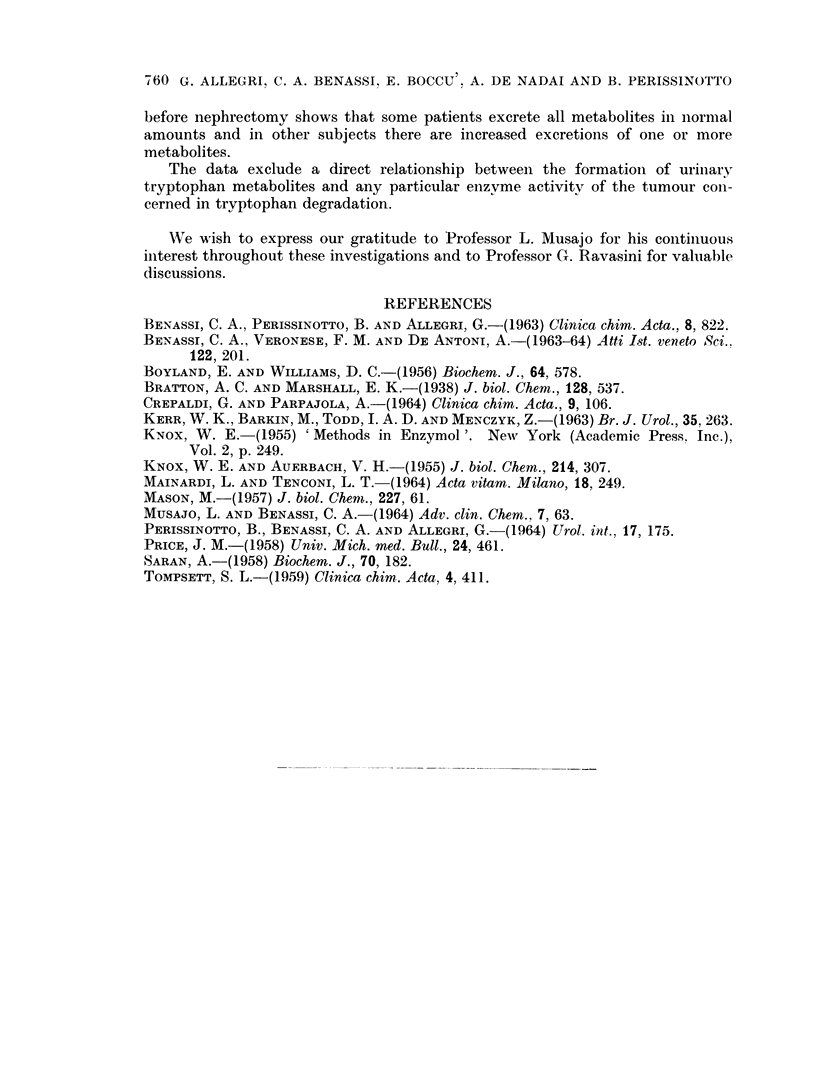# Tryptophan pyrrolase, kynureninase and kynurenine transaminase activities of human renal tumours.

**DOI:** 10.1038/bjc.1965.88

**Published:** 1965-12

**Authors:** G. Allegri, C. A. Benassi, E. Boccù, A. De Nadai, B. Persissinotto


### TRYPTOPHAN PYRROLASE, KYNURENINASE AND KYNURENINE TRANSAMINASE ACTIVITIES OF HUMAN RENAL TUMOURS*

G. ALLEGRI, C. A. BENASSI, E. BOCCU', A. DE NADAI AND B. PERISSINOTTO

*From the Institute of Pharmaceutical Chemistry, University of Padua, and the Urological Division of the Civic Hospital, Padua, Italy*

Received for publication June 16, 1965

IN preceding investigations (Benassi, Perissinotto and Allegri, 1963) we studied the excretion of tryptophan metabolites in 201 patients with bladder tumours, 55 subjects suffering from tumours of the urinary system, particularly of kidney (Perissinotto, Benassi and Allegri, 1964), and 112 individuals with various non-neoplastic urological diseases. Abnormal levels of urinary kynurenine, 3-hydroxy-kynurenine and 3-hydroxyanthranilic acid were detectable in certain patients in all three groups with a higher frequency in those with kidney neoplasm.

As a consequence of these results we doubted the relationship between the excretion of these metabolites and urinary tract neoplasm, and likewise it was difficult to accept the hypothesis that quantitative variations of metabolites, as revealed by increase of urinary products, could induce endogenous bladder cancer.

Since the presence of metabolites in biologic fluid depends upon specific enzyme activities we thought it of interest to study several of the enzymes involved in the intermediary metabolism of tryptophan.

In the present investigation tryptophan pyrrolase, kynureninase, and kynurenine transaminase were measured in normal and neoplastic human kidneys, since it was observed (Perissinotto *et al.*, 1964) that a higher and more frequent excretion of the so-called oncogenic metabolites occurred in patients with renal neoplasm.

Tryptophan pyrrolase firstly attacks tryptophan causing oxidative breakdown of the indole ring yielding $N^1$-formylkynurenine. It is present in mammalian, amphibian and avian liver and also in insects, some bacteria and fungi. Animal kidney seems to lack such an enzyme.

It has been isolated as ferriheme inactive protein and its levels in liver are under adaptive control : enzyme activity arises with increasing substrate concentration (induction by substrate) and furthermore by hormonal action (hormonal induction), and it is, in general terms, concerned with protein synthesis.

Kynureninase causes cleavage of the alanine chain of kynurenine and 3-hydroxykynurenine forming anthranilic and 3-hydroxyanthranilic acids respectively. It requires pyridoxal-5-phosphate as a coenzyme.

Kynurenine transaminase is also a pyridoxal-phosphate-dependent enzyme and catalyses the transformation of kynurenine to kynurenic acid and 3-hydroxy-kynurenine to xanthurenic acid.

* This paper was presented to the Symposium on Tryptophan Urinary Metabolites in London on May 17–19, 1965.

Mammalian liver has more kynureninase than kidney in which kynurenine transaminase is predominant although both enzymes are present in bacteria and moulds.

### MATERIALS AND METHODS

We have examined 23 renal parenchymal tumours (Table I) and 6 fragments of normal tissue from kidneys with a malignancy, since it was obviously impossible to have, as control, normal kidneys.

TABLE I.—*Number and Data of Kidney Tumours*

| No. | Case | Age | Sex | Diagnosis | Weight of tumour (g.) |
|---|---|---|---|---|---|
| 1 | V. A. | 64 | F. | Renal carcinoma | 22 |
| 2 | C. S. | 57 | M. | ,, ,, | 10 |
| 3 | G. B. | 67 | M. | ,, ,, | 9 |
| 4 | G. A. | 61 | F. | ,, ,, | 10 |
| 5 | G. A. | 60 | F. | ,, ,, | 222 |
| 6 | C. G. | 62 | M. | ,, ,, | 73 |
| 7 | A. M. | 55 | M. | ,, ,, | 94 |
| 8 | B. P. | 67 | M. | ,, ,, | 36 |
| 9 | M. V. | 58 | F. | ,, ,, | 170 |
| 10 | G. P. | 65 | M. | ,, ,, | 15 |
| 11 | M. M. | 56 | F. | ,, ,, | 20 |
| 12 | B. M. | 56 | M. | ,, ,, | 11 |
| 13 | G. R. | 54 | M. | Papillary adenocarcinoma | 46 |
| 14 | S. C. | 64 | F. | Renal carcinoma | 63 |
| 15 | O. R. | 47 | M. | ,, ,, | 26 |
| 16 | M. L. | 38 | M. | ,, ,, | 100 |
| 17 | B. V. | 45 | M. | ,, ,, | 120 |
| 18 | F. M. | 9 | F. | ,, ,, | 38 |
| 19 | R. F. | 18 | M. | ,, ,, | 18 |
| 20 | B. G. | 53 | M. | ,, ,, | 82 |
| 21 | M. T. | 67 | F. | ,, ,, | 43 |
| 22 | C. M. | 40 | M. | ,, ,, | 25 |
| 23 | C. A. | 53 | M. | ,, ,, | 60 |

Immediately after nephrectomy every organ was immersed in ice and divided into three portions, each of which was homogenized in a Waring Blendor for 2 minutes with 4 times its own weight of ice and phosphate buffer at a pH appropriate for each enzyme (see below). The homogenate was diluted to a 20% (w/v) concentration with 0·05 M buffer.

(1) Tryptophan pyrrolase was prepared and estimated according to Knox (1955) and to Knox and Auerbach (1955) with slight modifications. A 20% homogenate in 0·05 M phosphate buffer pH 7·0 was centrifuged for 10 minutes at 2500 r.p.m. and the supernatant used as the enzyme source.

The activity was determined for an incubation period of 60 minutes during which the reaction rate remained constant. Incubations were carried out in open Erlenmeyer flasks shaken in a water bath at 38° C. All assays were performed with an incubation mixture of 6·4 ml. of 0·1 M phosphate buffer, pH 7, 0·6 ml. of 0·03 M tryptophan in 0·1 M phosphate buffer, pH 7, 1 ml. of enzymic preparation.

Samples were run in duplicate and 1·0 ml. aliquots were removed at zero time for blanks and after 60 minutes of incubation and added to small tubes containing 1·0 ml. of 15 per cent trichloroacetic acid (TCA). The precipitated protein was centrifuged down for 10 minutes at 2000 r.p.m., and an aliquot of the clear supernatant was analysed for kynurenine content according the method of Bratton and Marshall (1938).

The amounts of kynurenine were estimated at 10–20 minute intervals for approximately two hours and the results plotted against time and hence it was possible to calculate the micromoles of kynurenine accumulating per hour for each gram of supernatant protein.

(2) Kynureninase was prepared and measured according to a modification of Saran's method (1958).

The frozen normal kidney and the neoplastic tissues were homogenized with cold 0·05 M phosphate buffer, pH 8, to prepare a 20 % mixture.

After freezing for a day at −20° C. it was thawed and centrifuged for 35 minutes at 9000 r.p.m. The assay mixture contained 1·0 ml. of supernatant fraction, 0·3 ml. of 0·00004 M pyridoxal-5-phosphate in 0·05 M phosphate buffer, pH 8, 1·6 ml. 0·05 M phosphate buffer, pH 8. After a 15 minute preincubation period at 37° C. 0·1 ml. of 0·01 M kynurenine in 0·05 M phosphate buffer, pH 8, was added.

From the incubation mixture were taken duplicate blanks of 1·2 ml. at zero time and text samples after 30 minute incubation.

The samples had 0·2 ml. of 15 % TCA added immediately to stop any enzyme reaction and were then centrifuged at 5000 r.p.m. for 10 minutes.

0·1 ml. supernatant was withdrawn and made to a final volume of 4 ml. with 0·1 M phosphate buffer, pH 8. The anthranilic acid formed was determined spectrofluorometrically with an Aminco-Bowman apparatus at 400 m$\mu$ (excitation 310 m$\mu$) and derived from the standard curve obtained in the same experimental conditions.

Enzyme specific activity was expressed as $\mu$moles of anthranilic acid formed per g. of protein per hour.

(3) Kynurenine transaminase was prepared and estimated by the method of Mason (1957) and was partially purified by means of ammonium sulphate fractionation.

All assays were performed in triplicate, with a 1·5 ml. mixture containing 1 ml. of enzyme solution, 0·15 ml. of 0·006 M $\alpha$-ketoglutarate in 0·05 M phosphate buffer, pH 7·4 and 0·15 ml. of 0·0004 M pyridoxal-5-phosphate in the same buffer.

After a 15 minute preincubation period at 37° C. 0·2 ml. of 0·035 M L-kynurenine in 0·05 M phosphate buffer, pH 7·4, was added. The reaction was stopped at zero time for blanks and after 30 minutes by means of 7·5 ml. of 95 % ethanol containing 1 % boric acid. Previous studies demonstrated a linear reaction rate during this time period.

After centrifuging at 9000 r.p.m. the formation of kynurenic acid was measured spectrophotometrically at 333 m$\mu$ against the blank containing the same components and deproteinizing agent added at zero time.

The amount of kynurenic acid formed was read from a calibration curve prepared under the same experimental conditions after adding known amounts of kynurenic acid.

Kynurenine transaminase activity was expressed as $\mu$moles of kynurenic acid formed per g. protein per hour.

(4) The nitrogen content of enzyme solutions was determined by the micro-Kjeldahl method.

(5) The 24 hours urines before nephrectomy were analysed for their content of tryptophan metabolites, by using the method of Benassi Veronese and Antoni (1963–64).

### RESULTS AND DISCUSSION

Table II reports data for enzymic activities : they are, to our knowledge, the first values on tryptophan pyrrolase, kynureninase and kynurenine transaminase in human renal tissue, from both normal and neoplastic kidneys.

TABLE II.—*Enzyme Activities of Normal and Neoplastic Kidneys*

Specific activities :

| Case number | Tryptophan pyrrolase* μM K/h./g. protein | | Kynureninase* μM AA/h./g. protein | | Kynurenine transaminase* μM KA/h./g. protein | |
|---|---|---|---|---|---|---|
| | Normal | Neoplastic | Normal | Neoplastic | Normal | Neoplastic |
| 1 | — | 0·6 | — | 0·00 | — | 87·6 |
| 2 | — | 0·0 | — | 0·22 | — | 18·4 |
| 3 | — | 0·8 | — | 0·00 | — | 3·6 |
| 4 | — | 0·6 | — | 0·20 | — | 39·4 |
| 5 | — | 0·0 | — | 0·06 | — | 0·9 |
| 6 | — | 0·8 | — | 0·25 | — | 5·8 |
| 7 | 0 | 1·2 | 0·17 | 0·23 | 2·9 | 2·0 |
| 8 | — | 0·0 | — | 0·30 | — | 2·1 |
| 9 | — | 0·0 | — | 0·29 | — | 11·9 |
| 10 | — | 0·0 | — | 0·65 | — | 2·7 |
| 11 | — | 0·0 | — | 0·38 | — | 25·2 |
| 12 | 0 | 0·0 | 0·15 | 0·25 | 8·5 | 7·0 |
| 13 | — | 0·0 | — | 0·21 | — | 3·1 |
| 14 | 0 | 0·0 | 0·54 | 0·78 | 5·3 | 6·8 |
| 15 | 0 | 0·0 | 0·22 | 0·00 | 4·3 | 3·8 |
| 16 | 0 | 0·0 | 0·31 | 0·04 | 21·2 | 0·0 |
| 17 | — | 0·0 | — | 0·00 | — | 2·8 |
| 18 | — | 0·0 | — | 0·00 | — | 0·0 |
| 19 | — | 0·0 | — | 0·69 | — | 6·5 |
| 20 | 0 | 0·6 | 0·33 | 0·14 | 0·0 | 5·8 |
| 21 | — | 0·0 | — | 0·00 | — | 0·0 |
| 22 | — | 0·0 | — | 0·39 | — | 1·9 |
| 23 | — | 0·0 | — | 0·45 | — | 8·9 |

* Values are expressed as an average of μmoles of compound formed.
The following abbreviations are used : K, kynurenine ; AA, anthranilic acid ; KA, kynurenic acid.

In this table one can note the absence of tryptophan pyrrolase in the normal kidney fragments. This confirms the absence of tryptophan pyrrolase in kidney tissue and shows a similar situation in renal neoplastic tissue.

Kynureninase values show large fluctuations both in normal and neoplastic kidney tissue. The activity does not significantly differ in normal and in oncogenic tissues. However, the activities are much lower than those obtained in this laboratory in analogous work on rat and guinea-pig kidney.

In the few cases in which it has been possible to isolate fragments of normal kidney, kynurenine transaminase results are slightly lower in the neoplastic portion than in the normal portion. However, the levels of this enzyme, somewhat higher than kynureninase, appear markedly reduced compared with that of other mammalian kidney when expressed as μmoles of product per g. protein per hour.

With an aim to relate the observed enzyme activities with the carcinogenic process the determination of main products of tryptophan degradation has been carried out in urine of the same patients 24 hours before nephrectomy and therefore just before the determination of kidney enzymic activities.

TABLE III.—*Amounts of Metabolites Excreted by 23 Subjects with Renal Tumour 24 hours Before Nephrectomy*

| Case No. | K | 3 0HK | 3 0HAA | oAHA | KA | XA |
|---|---|---|---|---|---|---|
| | | | mg./24 hr | | | |
| 1 | 2·10 | 0·58 | 1·80 | 0·87 | 1·32 | 0·86 |
| 2 | 1·09 | 0·30 | 0·26 | 0·52 | 1·80 | 0·60 |
| 3 | 0·89 | 0·25 | 0·27 | 0·63 | 0·90 | 0·56 |
| 4 | 1·10 | 0·50 | 0·44 | 0·63 | 1·08 | 0·50 |
| 5 | 0·92 | 0·58 | 0·32 | 0·58 | 1·88 | 0·72 |
| 6 | 22·50 | 0·52 | 0·88 | 0·42 | 0·95 | 0·52 |
| 7 | 15·00 | 0·49 | 0·40 | 0·48 | 2·80 | 0·66 |
| 8 | 1·10 | 0·28 | 25·11 | 0·82 | 1·92 | 0·52 |
| 9 | 0·97 | 0·36 | 0·32 | 0·75 | 1·40 | 0·50 |
| 10 | 17·50 | 0·49 | 7·60 | 1·20 | 8·38 | 7·62 |
| 10* | 17·50 | 0·28 | 6·38 | 0·97 | 7·57 | 7·24 |
| 11 | 0·92 | 0·26 | 0·32 | 1·32 | 0·95 | 0·48 |
| 12 | 0·98 | 0·42 | 8·69 | 0·63 | 0·97 | 0·50 |
| 13 | 1·22 | 0·40 | 0·68 | 0·97 | 1·20 | 0·52 |
| 14 | 68·75 | 0·50 | 2·50 | 1·52 | 2·85 | 0·70 |
| 15 | 2·20 | 0·80 | 0·88 | 35·42 | 12·58 | 5·26 |
| 16 | 1·20 | 0·49 | 0·60 | 0·88 | 1·00 | 0·60 |
| 17 | 1·14 | 0·52 | 0·42 | 0·40 | 2·82 | 0·72 |
| 18 | 37·50 | 0·30 | 23·18 | 0·58 | 1·20 | 0·60 |
| 19 | 12·50 | 0·65 | 0·32 | 0·92 | 8·62 | 0·72 |
| 20 | 20·00 | 0·50 | 3·50 | 1·20 | 1·40 | 0·86 |
| 21 | 1·28 | 0·28 | 0·40 | 0·82 | 1·80 | 0·60 |
| 22 | 1·10 | 0·32 | 0·66 | 0·58 | 0·95 | 0·70 |
| 23 | 1·20 | 0·40 | 0·80 | 0·82 | 1·00 | 0·72 |

The following abbreviations are used : K, kynurenine ; 3 0HK, 3-hydroxykynurenine ; 3 0HAA 3-hydroxyanthranilic acid ; *o*AHA, *o*-aminohippuric acid ; KA, kynurenic acid ; XA, xanthurenic acid.

* Values refer to urine directly collected from kidney which was the site of the tumour.

Underlined values refer to abnormally high excretion.

The data, reported in Table III, show that in some subjects the excretory pattern of all metabolites is perfectly normal before surgical excision of tumour.

Several workers (Boyland and Williams, 1956 ; Price, 1958 ; Tompsett, 1959 ; Mainardi and Tenconi, 1964 ; Musajo and Benassi, 1964) agree that all humans fed a free diet excrete daily small amounts of tryptophan metabolites. According to a method recently set up in this laboratory (Benassi *et al.*, 1963–64) the mean excretion of 20 normal individuals can be summarized as follows :

TABLE IV

| Metabolites | Average (mg./day) | (Range) |
|---|---|---|
| Kynurenine | 1·14 | (0·3–2·6) |
| 3-Hydroxykynurenine | 0·49 | (0·0–2·3) |
| Kynurenic acid | 2·83 | (1·0–4·2) |
| Xanthurenic acid | 0·66 | (0·3–1·8) |
| *o*-Aminohippuric acid | 1·14 | (0·4–1·7) |
| 3-Hydroxyanthranilic acid | 0·36 | (0·1–1·1) |

The comparison between Tables III and IV shows that in some kidney tumour patients there are levels of some metabolites grossly exceeding the normal values.

Cases No. 6, No. 7, No. 14, No. 19 and No. 20 excrete abnormally large amounts of kynurenine and cases No. 8 and No. 12 have excess of 3-hydroxyanthranilic acid and case No. 18 excretes high levels of 3-hydroxyanthranilic acid and kynurenine.

Case No. 15 shows exceptionally elevated levels of *o*-aminohippuric and also of kynurenic and xanthurenic acids.

In case No. 10 it was possible to examine urine passed per urethram and urine from the neoplastic kidney obtained through an ureteric catheter : both normal and neoplastic kidneys excrete daily the same quantities of metabolites and, except for 3-hydroxykynurenine and *o*-aminohippuric acid, in an abnormal manner. This finding is better and confirmatory evidence of the previous results that no relationship exists between metabolite excretion and kidney pathology and neither is there a relationship between the metabolites and the kidney's own enzymes.

Upon tryptophan loading in a patient with a small hypernephroma in the left kidney, Kerr *et al.* (1963) found, in contrast to the present result, that the " carcinogen " (i.e. 3-hydroxykynurenine and 3-hydroxyanthranilic acid) output and the ratio between " carcinogen " metabolites of tryptophan in different ureteric catheter collections over a fifteen minute period was 50 % more on the kidney with carcinoma. It should be noted that these results were obtained under different conditions to those presented here since our results relate only to spontaneous metabolite excretion.

The values obtained by us on the spontaneous excretion do not indicate that formation of tryptophan metabolites is attributable to enzyme activity of tumours, since we have demonstrated absence of tryptophan pyrrolase and comparable activities of kynureninase and kynurenine transaminase in normal and neoplastic kidney tissues. We think that these values for spontaneous metabolite excretion, when abnormal, are not ascribable to tumour enzymic activity.

Moreover one must remember that persistence of abnormal levels of urinary metabolites was found by us for periods of months and years after the excision of renal and bladder cancer.

Finally the more frequently occurring metabolites in abnormal amounts do not have the *o*-aminophenol structure and are not generally considered carcinogenic. An abnormal increase is however neither constant nor exclusive for urinary tract neoplasia. In fact, a similar situation occurs in persons with cancer in other sites and in all patients so far investigated with Hodgkin's disease (Crepaldi and Parpajola, 1964).

The data in the present investigation lead to the conclusion that tryptophan metabolites, when present in abnormal amounts in urine of kidney tumour patients, must not be regarded as derived from the tumour's own catabolism.

### SUMMARY

Tryptophan pyrrolase, kynureninase and kynurenine transaminase activities have been determined in 23 renal parenchymal tumours and, from 6 of these kidneys, normal tissue was obtained for comparison. Human kidney appears to lack tryptophan pyrrolase whilst kynureninase and kynurenine transaminase seem to have about the same activity in normal and neoplastic tissue.

The determination of tryptophan metabolites in urine collected 24 hours before nephrectomy shows that some patients excrete all metabolites in normal amounts and in other subjects there are increased excretions of one or more metabolites.

The data exclude a direct relationship between the formation of urinary tryptophan metabolites and any particular enzyme activity of the tumour concerned in tryptophan degradation.

We wish to express our gratitude to Professor L. Musajo for his continuous interest throughout these investigations and to Professor G. Ravasini for valuable discussions.

### REFERENCES

BENASSI, C. A., PERISSINOTTO, B. AND ALLEGRI, G.—(1963) *Clinica chim. Acta.*, **8**, 822.
BENASSI, C. A., VERONESE, F. M. AND DE ANTONI, A.—(1963–64) *Atti Ist. veneto Sci.*, **122**, 201.
BOYLAND, E. AND WILLIAMS, D. C.—(1956) *Biochem. J.*, **64**, 578.
BRATTON, A. C. AND MARSHALL, E. K.—(1938) *J. biol. Chem.*, **128**, 537.
CREPALDI, G. AND PARPAJOLA, A.—(1964) *Clinica chim. Acta.*, **9**, 106.
KERR, W. K., BARKIN, M., TODD, I. A. D. AND MENCZYK, Z.—(1963) *Br. J. Urol.*, **35**, 263.
KNOX, W. E.—(1955) 'Methods in Enzymol'. New York (Academic Press, Inc.), Vol. 2, p. 249.
KNOX, W. E. AND AUERBACH, V. H.—(1955) *J. biol. Chem.*, **214**, 307.
MAINARDI, L. AND TENCONI, L. T.—(1964) *Acta vitam. Milano,* **18**, 249.
MASON, M.—(1957) *J. biol. Chem.*, **227**, 61.
MUSAJO, L. AND BENASSI, C. A.—(1964) *Adv. clin. Chem.*, **7**, 63.
PERISSINOTTO, B., BENASSI, C. A. AND ALLEGRI, G.—(1964) *Urol. int.*, **17**, 175.
PRICE, J. M.—(1958) *Univ. Mich. med. Bull.*, **24**, 461.
SARAN, A.—(1958) *Biochem. J.*, **70**, 182.
TOMPSETT, S. L.—(1959) *Clinica chim. Acta*, **4**, 411.